# Web-Based Genome Analysis of Bacterial Meningitis Pathogens for Public Health Applications Using the Bacterial Meningitis Genomic Analysis Platform (BMGAP)

**DOI:** 10.3389/fgene.2020.601870

**Published:** 2020-11-26

**Authors:** Sean A. Buono, Reagan J. Kelly, Nadav Topaz, Adam C. Retchless, Hideky Silva, Alexander Chen, Edward Ramos, Gregory Doho, Agha Nabeel Khan, Margaret A. Okomo-Adhiambo, Fang Hu, Daya Marasini, Xin Wang

**Affiliations:** ^1^Laboratory Leadership Service Assigned to the National Center for Immunization and Respiratory Diseases, Centers for Disease Control and Prevention, Atlanta, GA, United States; ^2^Division of Bacterial Diseases, National Center for Immunization and Respiratory Diseases, Centers for Disease Control and Prevention, Atlanta, GA, United States; ^3^General Dynamics Information Technology, Contractor to Office of Informatics, Office of the Director, National Center for Immunization and Respiratory Diseases, Centers for Disease Control and Prevention, Atlanta, GA, United States; ^4^CDC Foundation Field Employee Assigned to Bacterial Meningitis Laboratory, Meningitis and Vaccine Preventable Diseases Branch, Division of Bacterial Diseases, National Center for Immunization and Respiratory Diseases, Centers for Disease Control and Prevention, Atlanta, GA, United States; ^5^Office of Informatics, Office of the Director, National Center for Immunization and Respiratory Diseases, Centers for Disease Control and Prevention, Atlanta, GA, United States; ^6^IHRC Inc., Contractor to Division of Bacterial Diseases, National Center for Immunization and Respiratory Diseases, Centers for Disease Control and Prevention, Atlanta, GA, United States; ^7^Weems Design Studio, Inc., Contractor to Division of Bacterial Diseases, National Center for Immunization and Respiratory Diseases, Centers for Disease Control and Prevention, Atlanta, GA, United States

**Keywords:** public health, genomics, bacterial meningitis, *Neisseria meningitidis* (meningococcus), *Haemophilus influenza*, bacterial capsule, molecular typing, serogroup B vaccine

## Abstract

Effective laboratory-based surveillance and public health response to bacterial meningitis depends on timely characterization of bacterial meningitis pathogens. Traditionally, characterizing bacterial meningitis pathogens such as *Neisseria meningitidis* (Nm) and *Haemophilus influenzae* (Hi) required several biochemical and molecular tests. Whole genome sequencing (WGS) has enabled the development of pipelines capable of characterizing the given pathogen with equivalent results to many of the traditional tests. Here, we present the Bacterial Meningitis Genomic Analysis Platform (BMGAP): a secure, web-accessible informatics platform that facilitates automated analysis of WGS data in public health laboratories. BMGAP is a pipeline comprised of several components, including both widely used, open-source third-party software and customized analysis modules for the specific target pathogens. BMGAP performs *de novo* draft genome assembly and identifies the bacterial species by whole-genome comparisons against a curated reference collection of 17 focal species including Nm, Hi, and other closely related species. Genomes identified as Nm or Hi undergo multi-locus sequence typing (MLST) and capsule characterization. Further typing information is captured from Nm genomes, such as peptides for the vaccine antigens FHbp, NadA, and NhbA. Assembled genomes are retained in the BMGAP database, serving as a repository for genomic comparisons. BMGAP’s species identification and capsule characterization modules were validated using PCR and slide agglutination from 446 bacterial invasive isolates (273 Nm from nine different serogroups, 150 Hi from seven different serotypes, and 23 from nine other species) collected from 2017 to 2019 through surveillance programs. Among the validation isolates, BMGAP correctly identified the species for all 440 isolates (100% sensitivity and specificity) and accurately characterized all Nm serogroups (99% sensitivity and 98% specificity) and Hi serotypes (100% sensitivity and specificity). BMGAP provides an automated, multi-species analysis pipeline that can be extended to include additional analysis modules as needed. This provides easy-to-interpret and validated Nm and Hi genome analysis capacity to public health laboratories and collaborators. As the BMGAP database accumulates more genomic data, it grows as a valuable resource for rapid comparative genomic analyses during outbreak investigations.

## Introduction

Rapid characterization of bacteria isolated from meningitis cases is critical for implementing successful public health responses and treatment strategies. *Neisseria meningitidis* [Nm] and *Haemophilus influenzae* [Hi], two important common causes of invasive bacterial meningitis worldwide, have traditionally been characterized by biochemical and molecular methods. Real-time PCR has been used to characterize Nm and Hi for clinical diagnosis of infection and surveillance purposes (Dolan Thomas et al., 2011; Wang et al., 2011b; Vuong et al., 2016). Sanger sequencing has been used for multilocus sequence typing (MLST) and fine typing of meningococcal isolates to establish clonal relationships between meningococcal strains and for typing of vaccine antigens to predict potential vaccine strain coverage (Maiden et al., 1998; Feavers et al., 1999; Birtles et al., 2005; Wang et al., 2011a). Biochemical and molecular laboratory tests can be time consuming and labor-intensive, but the recent proliferation of whole genome sequencing [WGS] technology has created an opportunity for streamlining the characterization of bacterial meningitis pathogens. Automated sequence analysis pipelines that identify the bacterial species (Topaz et al., 2018), Nm serogroup (Marjuki et al., 2019), and Hi serotype (Potts et al., 2019) provide a proof of concept for using WGS to elucidate bacterial meningitis pathogens.

Bioinformatic capability can be a bottleneck for laboratories without informatics expertise or for laboratories evaluating a diverse range of pathogens. These labs require WGS analysis pipelines that can quickly characterize sequencing data in a standardized manner. Additionally, WGS workflows for use in clinical and public health laboratories need to satisfy quality control metrics and produce validated results with acceptable accuracy and precision to ensure compliance with regulatory and clinical standards (Wong, 2013). Recently, PulseNet International was established to standardize laboratory-based surveillance for food-borne diseases using WGS (Nadon et al., 2017). Standardizing WGS analysis approaches ensures reproducible results among public health laboratories, invariably strengthening preparedness and reducing global, social and economic disease burden. To streamline and implement WGS analysis for bacterial meningitis pathogens, we developed the Bacterial Meningitis Genomic Analysis Platform [BMGAP]: a secure, web-accessible analytic and data management platform that provides an automated sequence analysis pipeline for Nm and Hi.

## Results and Discussion

### Genome Assembly and Quality Control

**FIGURE 1:**
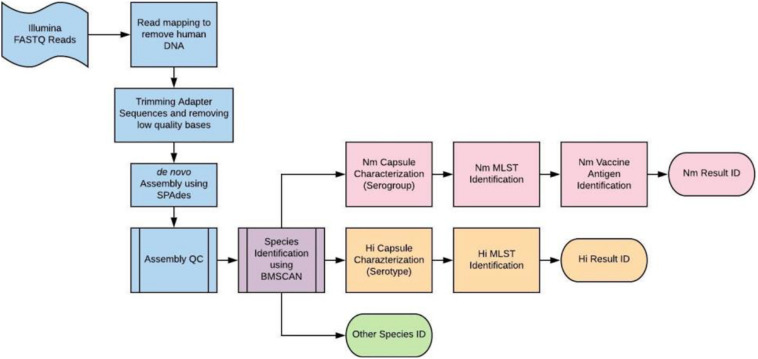
BMGAP single genome workflow.

BMGAP is currently designed for isolate sequencing analysis and takes short (e.g., 250 bp), high quality paired-end FASTQ read files as input. First, BMGAP processes FASTQ read files by trimming identified adapter sequences and removing low quality bases below Q of 20 using Cutadapt (Martin, 2011). The final trimmed, non-human reads are then used to generate *de novo* assembly using SPAdes (Bankevich et al., 2012). Next, each resulting assembly is assessed by average depth of coverage, as reported by the SPAdes assembler, and the evenness of coverage across contig. Assemblies are analyzed by three core modules: species identification, capsule characterization, and molecular typing (Topaz et al., 2018; Marjuki et al., 2019; Potts et al., 2019). The overall BMGAP workflow including each core module is illustrated in Figure 1. (For a summary of BMGAP’s QC parameters, see Supplementary Table 1).

### Data Transfer and Organization

BMGAP is a web application on the Office of Advanced Molecular Detection (OAMD) Portal, a genomics platform housed within the U.S. Centers for Disease Control and Prevention (CDC). For details on how to access BMGAP, please refer to https://github.com/CDCgov/BMGAP. Once a SAMS user account and password have been created, users can access BMGAP through the CDC OAMD portal gateway (CDC, 2020a,b). Upon accessing the OAMD portal, users can upload Illumina FASTQ files to be analyzed by BMGAP using the data transfer tool; data uploaded through the data transfer tool is recognized by BMGAP and automatically evaluated by the pipeline. Data processing and analysis lasts for a few hours to 1 day, depending on the size and complexity of the dataset.

**FIGURE 2:**
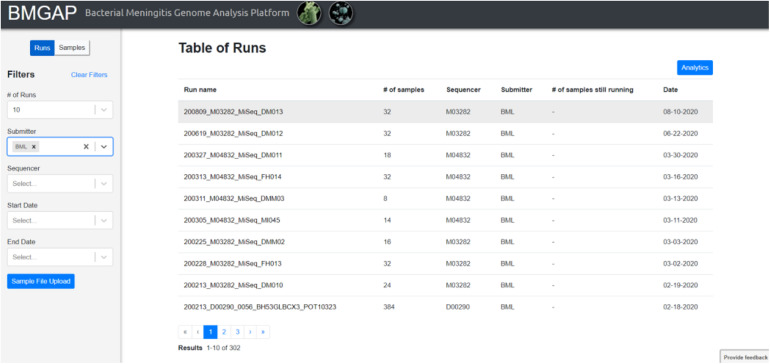
BMGAP online dashboard.

Upon completion of sequence analysis, users will see a list of runs representing sets of FASTQ files uploaded during a single session. Individual sample records can be viewed by clicking on the run or toggling the “Run/Samples” selection. Users can use the left dashboard to filter for specific sequences by submitter ID, sequencer, or a date range, or select isolates listed in a text document using the “Sample File Upload” button. The BMGAP interface is illustrated in Figure 2.

**FIGURE 3:**
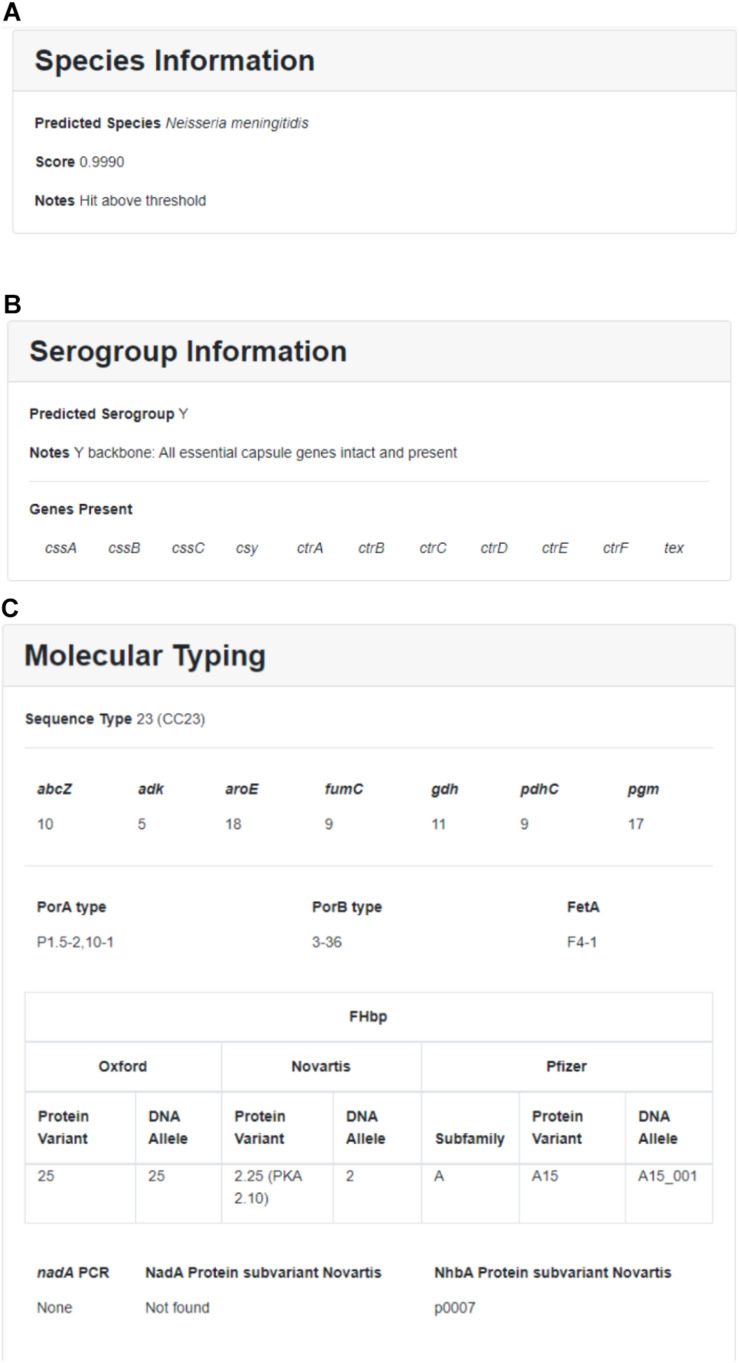
Sample results for individual Nm assembly featuring **(A)** species information, **(B)** Nm serogroup information, and **(C)** molecular typing.

Within each run, individual sequences are sorted by sample ID and can be further sorted by location, year, submitter, species, MLST, Nm serogroup, or Hi serotype. Individual sample reports can be viewed and printed by clicking on individual samples. The user can download detailed results and QC information for one or more samples by selecting the isolate(s) and clicking on the “Download” button. Downloaded results include additional information such as assembly statistics (e.g., assembly size, contig count, average depth of coverage, contigs with low depth of coverage), genome assembly (FASTA format), and genome annotation (GFF format). A sample report for one Nm genome assembly is illustrated in Figure 3.

### Genome Comparisons

**FIGURE 4:**
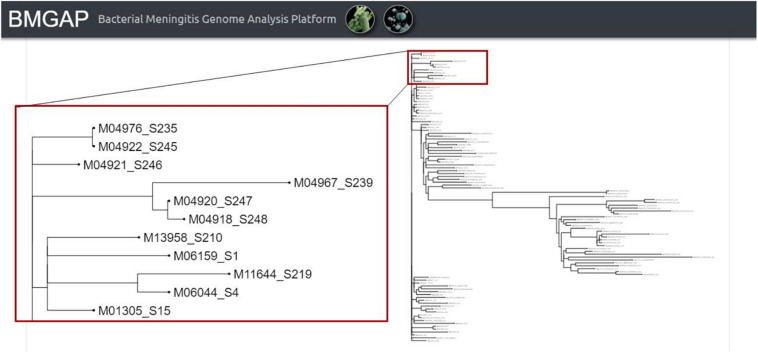
BMGAP phylogenetic trees.

Once the three core analysis modules have completed, users then have access to the comparative genomics module of BMGAP to further analyze their submitted sequences. Users can select specific genomes to compare against the BMGAP database to identify similar genomes using Mash, which quickly estimates genome similarity (Ondov et al., 2016). The relationships between these genomes can also be inferred by Neighbor Joining and visualized as a phylogenetic tree. An example of the phylogenetic tree is illustrated in Figure 4.

### Accuracy Evaluation

A total of 446 bacterial isolates submitted to the CDC Bacterial Meningitis Laboratory through surveillance programs between 2017 and 2019 were used to validate BMGAP’s performance. Each de-identified bacterial isolate came from a patient with invasive Nm or Hi disease, and the sample population is described in Table 1. The sample population consisted of the following isolates: 273 Nm isolates representing nine different Nm serogroups [A, B, C, E, W, X, Y, Z, and non-groupable (NG)] and 30 different clonal complexes; 150 Hi isolates representative of 7 different Hi serotypes [a, b, c, d, e, f, and non-typeable (NT)] and 54 different sequence types; and 23 isolates from nine other species that were neither Nm nor Hi. From this sample population, three Nm (NmA, NmC, NmNG) and three Hi (Hia, Hif, NTHi) strains were selected to be used as quality control (QC) strains to evaluate precision and depth of coverage (see Supplementary Table 2). The genomes were sequenced on an Illumina MiSeq to generate 250 bp paired-reads.

**TABLE 1:** Sample population for BMGAP method validation.

Organism	Number of isolates
*N. meningitidis* Serogroup A	2
*N. meningitidis* Serogroup B	42
*N. meningitidis* Serogroup C	31
*N. meningitidis* Serogroup E	4
*N. meningitidis* Serogroup W	22
*N. meningitidis* Serogroup X	4
*N. meningitidis* Serogroup Y	63
*N. meningitidis* Serogroup Z	2
*N. meningitidis*, Non-groupable	103
*H. influenzae* Serotype a	46
*H. influenzae* Serotype b	10
*H. influenzae* Serotype c	6
*H. influenzae* Serotype d	6
*H. influenzae* Serotype e	11
*H. influenzae* Serotype f	18
*H. influenzae*, Non-typeable	53
*Escherichia coli*	1
*Haemophilus haemolyticus*	3
*Haemophilus parainfluenzae*	2
*Neisseria bergeri*	5
*Neisseria gonorrhoeae*	4
*Neisseria lactamica*	1
*Neisseria polysaccharea*	1
*Neisseria subflava*	5
*Streptococcus pneumoniae*	1
Total	446

Overall accuracy, sensitivity, and specificity were 100% for Hi species identification, Hi serotype prediction, and Nm species identification. Overall accuracy, sensitivity, and specificity for Nm serogroup prediction were 99, 98, and 99%, respectively. For Nm serogroup prediction, BMGAP demonstrated high agreement with slide agglutination serogrouping (SASG; Cohen’s k = 0.95) with few disagreements. Intra-operator and intra-run precision were 100% (see Supplementary Tables 3, 4). The performance parameters for WGS are summarized in Table 2.

**TABLE 2:** Quality validation summary.

		Previous results (no. of samples)		% Sensitivity (95% CI)	% Specificity (95% CI)	Overall% agreement (95% CI)	κ (95% CI)
		
		Pos	Neg				
*H. influenzae* species ID	Pos	150	0	100 (98–100)	100 (99–100)	100 (99–100)	–
	Neg	0	296				
*H. influenzae* serotype prediction^*a*^	Pos	97	0	100 (96–100)	100 (93–100)	100 (98–100)	–
	Neg	0	53				
*N. meningitidis* species ID	Pos	273	0	100 (99–100)	100 (98–100)	100 (99–100)	–
	Neg	0	173				
*N. meningitidis* serogroup prediction^*b*^	Pos	167	2	99 (96–100)	98 (93–100)	99 (96–100)	0.95 (0.92–0.98)
	Neg	2	102				
Other bacterial species ID	Pos	23	0	100 (85–100)	100 (99–100)	100 (99–100)	–
	Neg	0	423				

*^*a*^For H. influenzae serotype, the previous results are SAST: BMGAP serotype predictions are reported as Pos in the table if the isolate was serotypeable and the predicted serotype matched the SAST results. BMGAP serotype predictions are reported as Neg if they are non-typable or do not match SAST results. No discrepancies were observed between BMGAP and SAST. ^*b*^For N. meningitidis serogroup, the previous results are SASG: BMGAP serogroup predictions are reported as Pos in the table if the isolate was serogroupable and the prediction matched the SASG results. BMGAP serogroup predictions are reported as Neg if they are non-groupable or do not match SASG results. Discrepancies are described in Table 3.*

Four discrepancies were observed between BMGAP and SASG. Two of these discrepant isolates were identified as NmY by BMGAP and NmW by SASG. For the first isolate, BMGAP detected an intact NmY polymerase gene (*csy*) and this isolate belonged to a clonal complex associated with NmY; however, the *csy* gene was not detected by PCR, possibly as a result of primers failing to amplify *csy*. The second isolate produced a serogroup W capsule despite having all NmY capsule genes intact and present by PCR and BMGAP. These two discrepancies between genogroup and capsule phenotype could be a result of cross-detection by antisera. The third discrepant isolate was identified as genogroup Y by BMGAP but NmE by SASG. After repeating SASG, this isolate did not agglutinate NmY antiserum, but displayed polyagglutination with the NmE antiserum. The last discrepant isolate was identified as genogroup Y by BMGAP and PCR, but NmNG by SASG. After repeating SASG, this isolate remained non-reactive with NmY antiserum. It is possible that the expression of NmY capsule in these two isolates could be lower than the visual detectable range for SASG given that all of the genetic elements required for expression of the NmY capsule are present according to WGS and PCR. Another consideration for these discrepancies could be heterogeneous NmY cultures in which reversible mutations such as internal stops could cause polyagglutination or no agglutination in SASG while genotypically being detected as NmY by WGS and PCR (Marjuki et al., 2019). Discrepancy resolution data is summarized in Table 3.

**TABLE 3:** Assay results for discordant samples.

Sample ID	BMGAP result	Previous SASG result	Repeat SASG result	Agree? (Y/N)	Comments
M42598	NmW	NmNG	NmW	Y	Intact NmW backbone; strong reaction with NmZ antisera
M43767	NmY	NmNG	NmY	Y	Intact NmY backbone; strong reaction with NmY antisera
M45194	NmY	NmNG	NmY & NmW	Y	Intact NmY backbone; strong reaction with NmY and NmW antisera
M42298	NmY	NmNG	NmY	Y	Intact NmY backbone; strong reaction with NmY antisera
M44887	NmZ	NmNG	NmZ	Y	Intact NmZ backbone; delayed reaction with NmZ antisera
M44113	NmNG	NmE	NmNG	Y	Intact NmZ backbone; non-specific weak reaction with all antisera
M43843	NmNG	NmW	NmNG	Y	NmB backbone with 1 internal stop and 1 missing codon; weak reaction with NmE antisera
M29998	NmY	NmNG	NmNG	N	Intact NmY backbone; no agglutination with any antisera
M44111	NmY	NmE	NmNG	N	Intact NmY backbone; weak reaction with NmE antisera
M46098	NmY	NmW	NmW	N	Intact NmY backbone; strong reaction with NmW antisera
M44738	NmY	NmW	NmW	N	Intact NmY backbone; strong reaction with NmW antisera

One limitation of our accuracy evaluation study is that BMGAP has been optimized for paired-end short reads as produced by Illumina sequencing and currently does not accept genome assemblies as input. This limitation does not pose an issue to public health and research laboratories that routinely perform Illumina sequencing, and can be accommodated depending on the future needs of these laboratories. As such, BMGAP will only perform read/assembly QC if sequencing reads are uploaded and BMGAP performs the assembly. By design, BMGAP is a surveillance tool and any additional analyses (e.g., antibiotic resistance gene detection) would require validation before being incorporated into the system. Once validated, additional BMGAP modules can be used as a research tool for research groups. Lastly, the BMGAP phylogeny is based on neighbor-joining of the rapid genetic distance estimates produced by Mash. Efforts are underway to standardize maximum likelihood phylogenetic analysis for incorporation, and optimizing it for rapid, automated results.

### Depth of Coverage Effect on Genome Assembly

Low depth of coverage can prevent the detection of genes and their correct sequences. We evaluated the minimum depth of coverage required for accurate assembly of Nm and Hi by providing the BMGAP pipeline with random subsets of reads drawn from the combined set of reads generated by three sequencing runs of the QC strains discussed previously. An accurate genome assembly was defined as the correct species, capsule type prediction, and molecular typing results (MLST genes for both species, plus FetA, PorA, NadA, FHbp, and NhbA for Nm). A preliminary evaluation was performed with a wide range of coverage values followed by a more extensive evaluation of the coverage range to determine the lowest depth of coverage where accuracy remained 100%. We calculated the assembly N50 as the length of the smallest contig in the set that contains the fewest (largest) contigs whose combined length represents at least 50% of the assembly (Miller et al., 2010).

**FIGURE 5:**
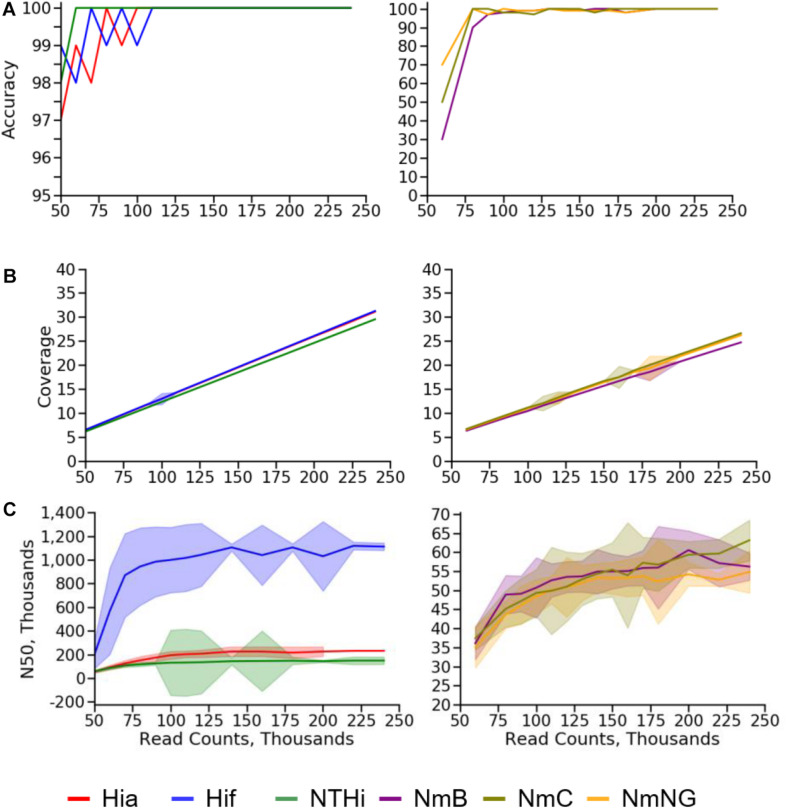
Subsampling results for **(A)** accuracy, **(B)** coverage, and **(C)** N50 values for Nm (right) and Hi (left) quality control strains.

As expected, the calculated depth of coverage had a strong linear relationship with the number of reads provided to the BMGAP pipeline. The lowest mean coverage value that produced no errors (100% accuracy) in species identification, capsule type prediction, or molecular typing results among Nm assemblies was 20X. NmC genomes with a depth of coverage of 20X had a lower N50 (53,776 bp) compared with NmB (60,549 bp) and NmNG (56,749 bp). The lowest mean coverage value that produced no errors in Hi assemblies was 14X. Hif genomes with a depth of coverage of 14X had a substantially higher N50 (1,016,958 bp) compared with Hia (202,788 bp) and NTHi (135,134 bp). The N50 data indicate that assembly quality degrades at lower depth of coverage. The subsampling results for accuracy, coverage, and assembly N50 are illustrated in Figure 5. A complete subsampling dataset is listed in Supplementary Tables 5, 6.

The high correlation between the number of read pairs and the depth of coverage reported by the SPAdes assembler enables depth of coverage to be used as a quality control statistic for assessing genome assembly quality. Nm and Hi genome assemblies with a depth of coverage over 20X never contained errors, indicating that 20X is a practical threshold for identifying genome assemblies that provide reliable molecular typing results and capsule type prediction. N50 values only increased modestly above this 20X threshold, indicating that using a higher threshold for quality control would raise sequencing costs but add little value. The high variability of N50 between genomes from the same species (especially *H. influenzae*) prevents this statistic from being used during routine quality control to assure the accuracy for molecular typing results and capsule type predictions.

Based on these findings, we would recommend using N50 in conjunction with depth of coverage and evidence of contamination to monitor for quality assurance. The quality strains will be regularly re-sequenced to assure that the pipeline continues to provide accurate results. Quality assurance monitoring will be conducted for every sequencing run to track the performance of the BMGAP over time. Validation of the pipeline will be repeated whenever new analysis modules are incorporated into BMGAP.

## Conclusion

The demand for standardized WGS analysis pipelines is increasing as more clinical and public health laboratories begin implementing WGS. We developed an informatics platform that provides automated bioinformatics tools available to public health laboratories and academic research laboratories wishing to characterize bacterial meningitis pathogens. BMGAP has demonstrated high accuracy and precision for species identification of several bacterial meningitis pathogens as well as serogroup and serotype prediction for Nm and Hi. Further, BMGAP establishes the infrastructure for developing and expanding an automated, multi-species analysis pipeline for bacterial meningitis pathogens and other pathogens of public health concern.

In recent studies, WGS was used to identify the possible origins of 15 epidemiologically distinct meningococcal outbreaks in the United States (Whaley et al., 2018), demonstrate the spread of a new hypervirulent strain of Nm (CC10217) in Africa (Bozio et al., 2018; Caugant, 2018), and monitor vaccine impact on Nm genetic diversity and strain replacement along the African meningitis Belt (Kretz et al., 2016; Retchless et al., 2016; Sidikou et al., 2016). These are examples of how WGS can be used to provide important information on how bacterial meningitis pathogens are disseminated in the population. By making BMGAP available through a secure online portal, users external to the CDC have access to these WGS-based analyses and can expand their capacity for analyzing Nm and Hi pathogens for rapid decision-making during meningitis outbreaks or for retrospective analyses.

BMGAP provides an automated platform for non-bioinformaticians in clinical and public health microbiology laboratories to obtain results that would typically require specialized bioinformatics knowledge. BMGAP will be expanded in the future to include identification of additional bacterial species and incorporate MLST for pathogens of public health concern. Generating this information enables laboratorians and public health professionals to link whole-genome sequencing data with epidemiological information which can enhance population-based surveillance in local and national health jurisdictions.

## Methods

### Whole Genome Sequencing

All isolates used for performance evaluation were extracted using the Chemagic Prepito (Perkin Elmer, Waltham, MA). Extracted DNA was assessed for quality using the NanoDrop 8000 and Qubit 2.0 Fluorometer (Thermo Fisher Scientific, Waltham, MA). Fragmentation of gDNA and library preparation was performed using the M220 Ultrasonicator (Covaris, Woburn, MA) and Zephyr Workstation (Caliper Life Science, Hopkinton, MA). Genomic libraries were assessed for quality using the 2200 TapeStation (Agilent Technologies, Santa Clara, CA) and Qubit 2.0 Fluorometer. Sequencing was performed using the MiSeq (Illumina, San Diego, CA), and the 250 bp, paired-end FASTQ files were uploaded to BMGAP for characterization. All commercial assays were performed according to the manufacturer’s instructions.

For accuracy evaluation, WGS results were compared with Nm slide agglutination serogrouping (SASG), Hi slide agglutination serotyping (SAST), and real-time polymerase chain reaction (rt-PCR). SASG and SAST were performed using commercially available antisera (Becton, Dickinson and Company, Franklin Lakes, NJ; Thermo Fisher Scientific, Waltham, MA). rt-PCR was performed as previously described (Dolan Thomas et al., 2011; Wang et al., 2011b, 2012; Vuong et al., 2016). For precision evaluation, two operators sequenced three independently generated libraries for three Nm (NmA, NmC, NmNG) and three Hi (Hia, Hif, NTHi) strains selected to be used for quality control (QC) (see Supplementary Table 2). Statistical analyses were conducted using Stata/IC version 14.

### Genome Analysis

During FASTQ read processing, common contaminants such as human, sheep, and PhiX sequences were removed by mapping reads to the hg38 human reference genome, the sheep genome, and PhiX genome using Bowtie2 v2.2.9 (Langmead and Salzberg, 2012). Following *de novo* assembly with SPAdes v3.13 (Bankevich et al., 2012), contigs with depth of coverage less than one-tenth of the genome-wide average are considered spurious and removed from the assembly whereas contigs with less than half of the genome-wide average coverage are considered potential contaminants. Assemblies with greater than 5% of their total size found in contigs with less than half of the genome wide depth of coverage are flagged for resequencing unless removal of spurious contigs brings the final amount to <1%.

The bacterial species was determined by comparing the genome assembly with a curated reference collection of 17 focal species with established similarity threshold values using BMSCAN as described previously (Topaz et al., 2018). If the query species was not one of the focal species, it is compared against the NCBI RefSeq collection (Pruitt et al., 2007) and the top match is reported with an indication that the species call has not been verified. Assemblies determined to be a species belonging to the *Neisseria* genus or Hi are characterized further by annotating genetic features. This is performed by comparing the sequences against a reference sequence database consisting of the PubMLST *Neisseria* and Hi allele collection supplemented with custom features such as insertion elements (Siguier et al., 2006, 2012; Kichenaradja et al., 2010) and other genes of interest using BLAST + (Camacho et al., 2009). After the genetic features are identified for these assemblies, the results are scanned to identify any capsule genes present, and these genes are used to describe the capsule genotype and predict the capsule phenotype of the pathogen, as previously described (Marjuki et al., 2019; Potts et al., 2019).

The molecular typing module consists of comparing MLST, fine typing and vaccine antigen alleles against defined loci schemes provided by PubMLST using BLAST + v2.2.30 (Camacho et al., 2009). Multi-Locus Sequence Typing (MLST) is reported for both Nm and Hi genomes. For Nm, Porin A (PorA), Porin B (PorB), and Ferric enterobactin transport (FetA) types are defined by their respective variable regions (Jolley et al., 2018). *Neisseria* adhesin A (NadA) is identified by variant and peptide ID, as suggested by Bambini et al. (2014). Both *Neisseria* Heparin Binding Antigen (NhbA) and Factor H binding protein (FHbp) are identified by their PubMLST peptide identifiers.

To artificially generate test sets with low depth of coverage (subsampling), we concatenated FASTQ read files generated from three independently prepared libraries for each of the six QC strains discussed previously (see Supplementary Table 2), then randomly sampled reads using seqtk version 1.0-r31 (Li, 2020), using a different random seed for each replicate. The preliminary test covered a broad range of values from 6 × 10^4^ to 24 × 10^4^ read pairs (3 × 10^7^ to 12 × 10^7^ nucleotides) with 10 replicates. Results from the preliminary test revealed decreased accuracy with fewer than 1 × 10^5^ read counts for Hi (∼15X coverage) and 2 × 10^5^ read counts for Nm (∼20X coverage). A full evaluation was performed with 100 replicates for each QC strain, using 5–12 × 10^4^ read pairs (2.5–6 × 10^7^ nucleotides) for Hi and 9–18 × 10^4^ read pairs (4.5–9 × 10^7^ nucleotides) for Nm. Eleven of the test sets produced small genome assemblies with low depth of coverage, resulting in a failure to produce serogroup/serotype predictions and molecular typing results (98.2% accuracy). Preliminary and full evaluation datasets are listed in Supplementary Table 4.

The BMGAP analysis pipeline runs on CDC’s Aspen high-performance computing cluster, which uses Univa Grid Engine as the job scheduler. The BMGAP web application is written in Javascript, with the React framework used to create the web interface and ExpressJS used to serve data from the MongoDB database where BMGAP results are stored. The phylogenetic tree visualizations generated by feeding Mash distance comparisons into the biopython phylo package (Talevich et al., 2012). Updates and version control of BMGAP and its submodules are managed as Git repositories with public releases hosted at https://github.com/CDCgov/BMGAP. A pipeline version identifier is stored with the analysis results and updates to the analysis pipeline will be tested on control datasets to assure consistency with previous versions. The overall system meets the E-Authentication Assurance Level 3 (EAAL) standard ensuring high confidence in system processing and storing sensitive data. Once user identity is verified and appropriate login credentials are created, all sequence information uploaded by the user or laboratory group is protected by a firewall and managed by individual groups. Members within a group can see all of the uploaded sequence data associated within the group, and users from external groups are restricted to limited information about individual sequences such as an arbitrary identification number and the year of analysis. Adherence to federal security and privacy standards ensure that sequence analysis activities conducted through the OAMD portal are secure and protect information.

## Data Availability Statement

The BMGAP source code for this study can be found in the git repository for BMGAP (https://github.com/CDCgov/BMGAP). BMGAP is an application made available through web portal for the Office of Advanced Molecular Detection (OAMD) from the U.S. Centers for Disease Control and Prevention (https://amdportal-sams.cdc.gov/portal/).

## Ethics Statement

This analysis of genomic and immunologic data was determined not to be human subjects research by the CDC National Center for Immunization and Respiratory Diseases (P_2017_DBD_Wang_411).

## Author Contributions

SB prepared the manuscript, evaluated performance, and conducted discrepancy resolution. RK developed the BMGAP system. AR and NT developed individual modules of BMGAP (e.g., locus extractor, BMSCAN). HS, AC, ER, and GD contributed to the BMGAP user interface, data management, and development support. AK, MO-A, GD, and XW supervised and directed the BMGAP development team. FH and DM served as the chief test operators for the BMGAP whole genome sequencing process. All authors provided critical review of the manuscript.

## Conflict of Interest

RK, HS, ER, and GD were employed by General Dynamics Information Technology. FH was employed by IHRC Inc. DM was employed by Weems Design Studio, Inc. The remaining authors declare that the research was conducted in the absence of any commercial or financial relationships that could be construed as a potential conflict of interest.
