# Supplementary material for: Web-Based Genome Analysis of Bacterial Meningitis Pathogens for Public Health Applications Using the Bacterial Meningitis Genomic Analysis Platform (BMGAP)
Source: Front Genet. 2020 Nov 26;11:601870. doi: 10.3389/fgene.2020.601870 (PMC7726215; doi:10.3389/fgene.2020.601870)
Supplement: Supplementary file 1 [file Data_Sheet_1.PDF]

Supplementary Material

Supplemental Table 1: Quality assurance metrics for BMGAP

Quality Metric	Threshold
Expected Genome Size	2.0-2.4 MB (Nm); 1.6-2.0 MB (Hi)
Average Depth of Coverage	20X
Discard spurious contig	Depth of coverage <1/10 of genome-wide average.

Supplemental Table 2: Quality control strains used to evaluate subsampling and precision.

BML ID Number	QC Organism	MLST Sequence Type
M05178	*N. meningitidis*, Serogroup B	32
M03045	*N. meningitidis*, Serogroup C	11
M05075	Nongroupable *N. meningitidis*	6804
M04741	*H. influenzae*, serotype a	56
M06297	*H. influenzae*, serotype f	124
M05209	Non-typeable *H. influenzae*	2

526 **Supplemental Table 3:** Intra-run and Intra-operator precision for *N. meningitidis* controls.

Run ID	Tech ID	Coverage Depth	Percent Discarded	Species ID	Capsule ID	Pass? Y/N
DM007	2	54.518	0.742	*N. meningitidis*	B	Y
DM008	2	63.533	0.488	*N. meningitidis*	B	Y
FH009	1	34.277	1.924	*N. meningitidis*	B	Y
FH011	1	37.398	0.742	*N. meningitidis*	B	Y
DM007	2	48.453	1.173	*N. meningitidis*	C	Y
DM008	2	48.980	1.023	*N. meningitidis*	C	Y
FH009	1	36.560	0.796	*N. meningitidis*	C	Y
FH011	1	40.742	1.173	*N. meningitidis*	C	Y
DM007	2	50.759	0.625	*N. meningitidis*	NG	Y
DM008	2	49.157	0.645	*N. meningitidis*	NG	Y
FH009	1	39.038	0.733	*N. meningitidis*	NG	Y
FH011	1	43.948	0.625	*N. meningitidis*	NG	Y

527 **Supplemental Table 3:** Intra-run and Intra-operator precision for *H. influenzae* controls.

Run ID	Tech ID	Coverage Depth	Percent Discarded	Species ID	Capsule ID	Pass? Y/N
DM007	2	79.151	1.233	*H. influenzae*	a	Y
DM008	2	65.190	3.198	*H. influenzae*	a	Y
FH009	1	60.106	1.000	*H. influenzae*	a	Y
FH011	1	65.402	0.891	*H. influenzae*	a	Y
DM008	2	83.981	4.153	*H. influenzae*	f	Y
DM007	2	75.688	1.191	*H. influenzae*	f	Y
FH009	1	57.718	1.124	*H. influenzae*	f	Y
FH011	1	51.467	0.761	*H. influenzae*	f	Y
DM007	2	77.416	1.701	*H. influenzae*	NT	Y
DM008	2	90.678	4.481	*H. influenzae*	NT	Y
FH009	1	55.231	1.115	*H. influenzae*	NT	Y
FH011	1	50.571	0.658	*H. influenzae*	NT	Y

This is a provisional file, not the final typeset article

Supplemental Table 4: Subsampling was performed in two different phases: 1) Preliminary evaluation was performed with 10 replicates for each control for a broad range of sequence dataset sizes to determine the lowest value with the no errors in test results (N); 2) Full evaluations were performed using 100 replicates for each control sampled in a range closer to the lowest value that had no errors in the preliminary test results.

Approximate Coverage for 2MB Genome	Preliminary Evaluation (n = 10 replicates)	Full Evaluation (n = 100 replicates)
**6-8X**	6×10^{4}	5×10^{4} (Hi only)
**8-10X**	8×10^{4}	7×10^{4} (Hi only)
**10-13X**	10×10^{4}	9×10^{4}
**12-15X**	12×10^{4}	11×10^{4}
**14-18X**	14×10^{4}	13×10^{4} (Nm only)
**16-20X**	16×10^{4}	15×10^{4} (Nm only)
**20-23X**	18×10^{4}	17×10^{4} (Nm only)
**22-26X**	20×10^{4}	-
**24-28X**	22×10^{4}	-
**25-32X**	24×10^{4}	-

Supplemental Table 5: Subsampling results for Hi (a) and Nm (b) quality control strains. Accuracy is the percentage of replicates that had no errors in species identification, capsule type prediction, or molecular typing; mean coverage is calculated from the average depth of coverage across replicates; mean N50 is the mean N50 across replicates. (*) indicates subsamples run in the preliminary dataset only. (†) indicates subsamples run in the preliminary and final dataset. (‡) indicates subsamples run in the final dataset only.

(a)

**Read Pair Count** (10^3)	**Replicates**	**QC *H. influenzae* serotype a**			**QC *H. influenzae* serotype f**			**QC *H. influenzae*, Non-typeable**		
		**Accuracy**	**Mean Coverage**	**Mean N50**	**Accuracy**	**Mean Coverage**	**Mean N50**	**Accuracy**	**Mean Coverage**	**Mean N50**
**50**‡	100	97%	6.54	55861	99%	6.55	201415	98%	6.20	58421
**60**†	110	99%	7.83	89690	98%	7.85	409257	100%	7.44	89495
**70**‡	100	98%	9.13	125569	100%	9.17	870428	100%	8.66	108224
**80**†	110	100%	10.43	150763	99%	10.47	1046998	100%	9.89	116684
**90**‡	100	99%	11.73	173933	100%	11.78	984728	100%	11.12	124910
**100**†	110	100%	13.03	201495	99%	13.09	971654	100%	12.35	132293
**110**‡	100	100%	14.33	202788	100%	14.39	1016958	100%	13.59	132631
**120**†	110	100%	15.64	191708	100%	15.70	1120542	100%	14.83	134024
**140***	10	100%	18.21	225615	100%	18.28	1105515	100%	17.27	143767
**160***	10	100%	20.81	225615	100%	20.89	1040432	100%	19.75	145395
**180***	10	100%	23.41	216093	100%	23.49	1105534	100%	22.17	147392
**200***	10	100%	25.99	225615	100%	26.09	1031111	100%	24.64	143774
**220***	10	100%	28.52	231242	100%	28.70	1118733	100%	27.10	149015
**240***	10	100%	31.13	231242	100%	31.27	1112566	100%	29.55	149015

This is a provisional file, not the final typeset article

(b)

Read Pair Count (10^3)	Replicates	QC *N. meningitidis* Serogroup B			QC *N. meningitidis* Serogroup C			QC *N. meningitidis, Nongroupable*		
		Accuracy	Mean Coverage	Mean N50	Accuracy	Mean Coverage	Mean N50	Accuracy	Mean Coverage	Mean N50
60[*]	10	30%	6.39	36109	70%	6.61	35133	50%	6.74	37412
80[*]	10	90%	8.49	48876	100%	8.80	43616	100%	8.97	45136
90[‡]	100	97%	9.52	49091	97%	9.88	46079	100%	10.07	47173
100[†]	110	98%	10.68	51564	100%	10.95	48795	98%	11.19	50097
110[‡]	100	99%	11.59	52620	99%	12.06	50096	98%	12.07	49882
120[†]	110	99%	12.82	57258	99%	13.12	51807	97%	13.42	52510
130[‡]	100	100%	13.66	53650	100%	14.25	51870	100%	14.50	52877
140[†]	110	100%	14.94	57100	99%	15.30	53465	100%	15.65	55330
150[‡]	100	99%	15.70	54962	99%	16.43	53177	100%	16.71	55418
160[†]	110	100%	17.06	55605	99%	17.47	53790	98%	17.89	55213
170[‡]	100	100%	17.73	55857	99%	18.60	53719	100%	18.93	57234
180[†]	110	98%	19.20	60509	98%	19.65	53776	100%	20.12	56749
200[*]	10	100%	20.75	60549	100%	21.86	54209	100%	22.26	59344
220[*]	10	100%	22.76	57132	100%	24.04	52758	100%	24.43	59626
240[*]	10	100%	24.75	56,221	100%	26.24	54855	100%	26.64	63142
